# Basophil Activation Test with Different Polyethylene Glycols in Patients with Suspected PEG Hypersensitivity Reactions

**DOI:** 10.3390/ijms232314592

**Published:** 2022-11-23

**Authors:** Simone Vespa, Pietro Del Biondo, Pasquale Simeone, Enrico Cavallucci, Giulia Catitti, Raffaella Auciello, Domenico De Bellis, Isotta Altomare, Laura Pierdomenico, Barbara Canonico, Ilaria Cicalini, Ilaria Angilletta, Piero Del Boccio, Damiana Pieragostino, Francesca Santilli, Andrea Urbani, Vincenzo De Laurenzi, Liborio Stuppia, Paola Lanuti

**Affiliations:** 1 Department of Medicine and Aging Sciences, University “G. d’Annunzio” of Chieti-Pescara, 66100 Chieti, Italy; 2 Center for Advanced Studies and Technology (CAST), University “G. d’Annunzio” of Chieti-Pescara, 66100 Chieti, Italy; 3 Scuola di Specializzazione in Allergologia e Immunologia Clinica, University “G. d’Annunzio” of Chieti-Pescara, 66100 Chieti, Italy; 4 Allergology Unit, ASL2, Chieti-Lanciano-Vasto, 66100 Chieti, Italy; 5 Clinical Pathology Unit, ASL2, Chieti-Lanciano-Vasto, 66100 Chieti, Italy; 6 Department of Biomolecular Sciences, University of Urbino Carlo Bo, 61029 Urbino, Italy; 7 Department of Pharmacy, University “G. d’Annunzio” of Chieti-Pescara, 66100 Chieti, Italy; 8 Department of Innovative Technologies in Medicine and Dentistry, University “G. d’Annunzio” of Chieti-Pescara, 66100 Chieti, Italy; 9 Dipartimento di Chimica Biochimica e Biologia Molecolare Clinica, Fondazione Policlinico Universitario A. Gemelli I.R.C.C.S., 00168 Rome, Italy; 10 Faculty of Medicine and Surgery, Università Cattolica del Sacro Cuore, 00168 Rome, Italy; 11 Department of Psychological, Health and Territorial Sciences, School of Medicine and Health Sciences, University “G. d’Annunzio” of Chieti-Pescara, 66100 Chieti, Italy

**Keywords:** allergy testing, COVID-19, hypersensitivity, polyethylene glycol, basophil activation tests, stimulation index

## Abstract

Allergic reactions to COVID-19 vaccine components are rare but should be considered. Polyethylene glycol (PEG) is responsible for anaphylaxis in mRNA vaccines. Skin tests have been used in the allergological work-up programs for COVID-19 vaccine evaluation. However, the reproducibility of the skin prick test is time-dependent and the reactivity declines over time. Therefore, we combined the administration of the skin tests with the basophil activation test (BAT) using PEG2000, PEG4000 and DMG-PEG2000, where the BAT was considered positive when the percentage of activated basophils was higher than 6%, 5% and 6.5%, for PEG 4000, PEG2000 and DMG-PEG2000, respectively. To this end, among the subjects that underwent allergy counseling at the Allergy Unit of our Institution during the 2020/2021 vaccination campaign, 13 patients had a suggested medical history of PEG/drug hypersensitivity and were enrolled together with 10 healthy donors. Among the enrolled patients 2 out of 13 tested patients were positive to the skin test. The BAT was negative in terms of the percentages of activated basophils in all analyzed samples, but the stimulation index (SI) was higher than 2.5 in 4 out of 13 patients. These data evidenced that, when the SI is higher than 2.5, even in the absence of positivity to BAT, the BAT to PEG may be a useful tool to be coupled to skin tests to evidence even low-grade reactions.

## 1. Introduction

The pandemic declared in March 2020 by the World Health Organization (WHO) and induced by severe acute respiratory syndrome coronavirus-2 (SARS-CoV-2) produced an unprecedented challenge for worldwide healthcare systems [1]. The development, in a short time, of new vaccines to prevent SARS-CoV-2 infection was one of the most relevant scientific landmark achievements in this context [2,3]. Despite the administered COVID-19 vaccination platforms ensuring the development of specific humoral and cellular immune responses, protecting against severe disease symptoms [4,5,6,7], several adverse effects were reported, even if most of them were classified as mild reactions [8]. Furthermore, in most patients reporting adverse effects, no events occurred after a second vaccination, demonstrating that most of the reported reactions were not allergic events but vasovagal and anxiety signs. Nevertheless, hypersensitivity reactions were reported in a small number of subjects, and anaphylaxis was confirmed in 0.027% and 0.023% of individuals who received the Pfizer/BioNTech BNT162b2 and Moderna mRNA-1273 vaccines, respectively [9]. Overall, 0.06 fatal anaphylaxis cases per 10^6^ doses of COVID-19 vaccine were registered, indicating that fatal events are extremely rare [8], but the echo from social media resulted in public distress and in a loss of confidence in the safety of the vaccination campaign. Therefore, the European Academy of Allergy and Clinical Immunology (EAACI) evaluated all vaccine components for the related allergenic potential [10]. Polyethylene glycol (PEG) and polysorbate 80 (PS80) were therefore identified as causal allergens contained in mRNA and viral vector-based vaccines, respectively [2,11,12,13,14,15,16]. Polysorbate 80 (PS80) is a non-ionic detergent used to solubilize proteins and is present in viral vector COVID-19 vaccines (i.e., Janssen/J&J Ad26.COV2.S and Oxford/AstraZeneca ChAdOx1-S) [17]. However, mRNA vaccines contain the mRNA encoding for the full-length SARS-CoV-2 spike (S) protein [18], protected and encapsulated within lipid nanoparticles (LNPs). A process known as PEGylation is used for the coating of PEGs on LNP surfaces, to reduce opsonization, aggregation, and improve mRNA delivery to the target cells, but at the same time, this process alters the immunogenic properties of PEGs [19]. It has been demonstrated that PEGylated liposomes, but not PEG alone without lipid conjugation, induced a relevant basophil activation in a dose-dependent manner in patients with a history of PEG allergy [19]. Both the Moderna mRNA-1273 and Pfizer/BioNTech BNT162b2 vaccines use PEG/lipid conjugates as a part of the LNPs; while the mRNA-1273 vaccine contains DMG-PEG 2000 (formed by the PEGylation of the lipid dimyristoyl glycerol), the BNT162b2 vaccine uses PEG-2000 as a PEG/lipid conjugate [20,21]. Little is known about the mechanisms for the induction of anaphylaxis; contact system activation by nucleic acids, complement recognition of the vaccine-activating allergic effector cells, direct mast cell activation, and pre-existing antibody recognition of PEG were all action mechanisms potentially associated with COVID-19 vaccine administration in allergic patients [22].

In any case, regulatory agencies established specific recommendations for the safe administration of COVID-19 vaccines based on the review of the allergic adverse reactions potentially occurring after vaccination [1,23]. The allergological work-up programs for the evaluation of the allergic potential of allergens contained in COVID-19 vaccines were mainly focused on PEGs, given that in Italy, as in many other countries, the viral vector-based vaccines were withdrawn from the market [24]. For these reasons, the most widely used allergological work-up program for the evaluation of COVID-19 vaccine allergies included the administration of a skin prick test (SPT) with PEG-containing products; an intradermal test (IDT) and sIgE evaluation were also applied [17,25,26]. Notably, it has been demonstrated that, in this context, the reproducibility of the SPT test is time-dependent and reactivity will decline over time [24]. However, when associated with the basophil activation test (BAT), the SPT gave better results [27]. Even if BAT was not largely applied during the COVID-19 vaccination campaign, it has great potential in this sense [28]. The BAT is based on the flow cytometry evaluation of the CD63 expression on basophil cell surfaces. The glycoprotein CD63 is exposed on the basophil surface granules, and, upon basophil activation, it becomes expressed on the basophil surface, acting as a basophil activation marker [29]. 

Here we implemented the BAT in a cohort of patients that reported a previous allergic reaction to vaccines and/or PEGs, to determine the value of such a test in the allergological work-up program for patients at risk to develop allergic reactions to COVID-19 vaccine components. 

## 2. Results

### 2.1. Skin Test Analyses

During the vaccination campaign, in the area covered by the Allergy Unit of the “SS Annunziata’’ Hospital of Chieti, 822,998 doses of COVID-19 vaccines were administered, with 296,143 fully vaccinated people (at least two doses). More than 800 patients with a history of severe drug reactions, including vaccines, underwent allergy counseling, as established by the shared protocol for allergic patients in the Chieti-Lanciano-Vasto ASL. Among 61 patients with a suspected PEG hypersensitivity, 13 of them were selected and tested with PEG-SPT and IDT and 2 out of 13 of them showed a detectable positivity to the PEG-SPT or IDT (Table 1). The BAT was carried out as a third-level test since, as reported in the literature, PEG-SPT and IDT showed very poor sensitivity in patients with a suggestive clinical history but were negative for the skin tests.

### 2.2. Basophil Activation Test Analyses

**Figure 1:**
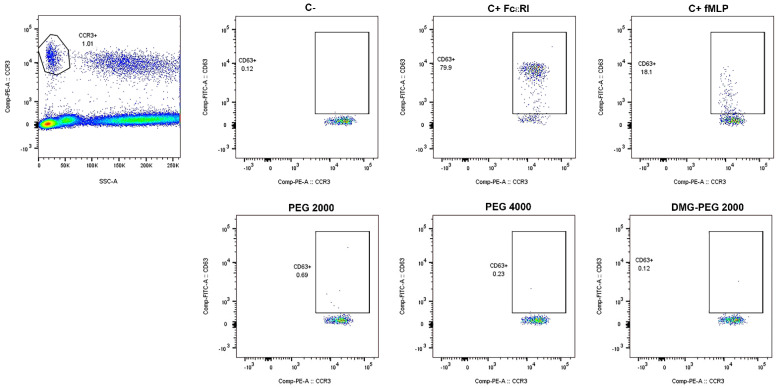
Gating strategy. SSClow/CCR3+ events were firstly selected on an SSC-A/CCR3-PE dot-plot, and then CD63-positive basophils were identified on a CCR3-PE/CD63-FITC dot-plot for the negative control (C−), positive controls (C+ FcεRI and C+ fMLP) and allergens (PEG 2000, PEG 4000 and DMG-PEG 2000). The gating strategy is representative of all analyzed samples.

As immunosuppressors, including corticosteroids, potentially affect BAT results [30], all enrolled subjects underwent an immunosuppressor 48 h washout, as recommended, to avoid potential confounding effects. In such a context, the identification of activated basophils was based on the flow cytometry detection of CD63 as a basophil activation marker. The applied gating strategy, reproducing previously published indications [31], is fully shown in Figure 1. Briefly, CCR3-positive events were firstly selected on an SSC-A/CCR3-PE dot-plot, and then CD63-positive activated basophils were identified on a CCR3-PE/CD63-FITC dot-plot. The protocol was applied to the peripheral blood of 13 patients and 10 healthy volunteers and PEG2000 (for testing BNT162b2), PEG4000, and DMG-PEG2000 (for mRNA-1273) were used for basophil stimulation. Neither patients nor healthy volunteers, after the stimulation, showed a percentage of activated basophils (CCR3+/CD63+ cells) above the threshold (6%, 5% and 6.5%, for PEG 4000, PEG2000 and DMG-PEG2000, respectively), for none of the used PEGs (Tables S1 and S2). As they did not exhibit any significant activation when stimulated with the positive controls, two patients were classified as non-responders (NR, Table S1).

### 2.3. The Value of the Stimulation Index for the BAT Evaluation

**Figure 2:**
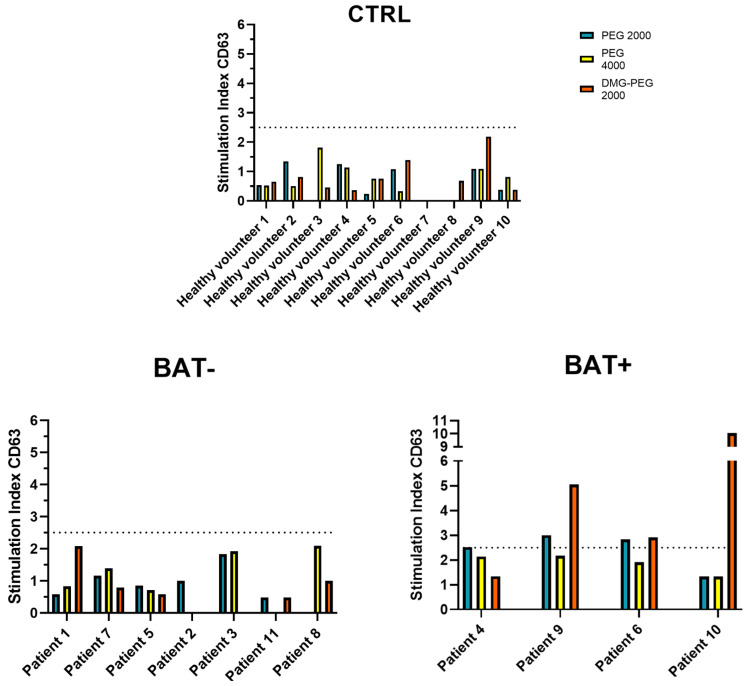
Stimulation index (SI) of CD63 expression. The graphs show the SI of the control group, patients that were negative for the test (BAT−), and patients that were considered positive (BAT+). SI > 2.5 was considered as positivity for the test.

As suggested for other medications, positivity to the BAT can be expressed as SI values [32]. For this reason, even if the BAT was negative for all patients, the SI was calculated for all BAT-analyzed patients, and it emerged that 36.36% of responder patients (4 out of 11 patients) showed a SI value > 2.5 (Figure 2) for at least one of the tested PEGs (PEG4000, PEG2000 or DMG-PEG2000). When the percentages of activated basophils and the SI of the healthy volunteers and patients were compared, only the PEG2000 SI was significantly higher in patients than in healthy volunteers (Tables S2 and S3). Of note, none of the healthy volunteers displayed SI values > 2.5 (Figure 2). Notably, it has been demonstrated that, when percentages of activated basophils are considered, a positive result in the BAT may indicate a past COVID-19 infection instead of an allergy [28]. It must be underlined that none of the control subjects displayed positivity to tested PEGs, even if 3 out of 10 of them (healthy volunteers 1, 4, and 6, Figure 2) experienced a past COVID-19 natural infection.

### 2.4. Evaluation of Skin Test and BAT Analyses

Table 1 summarizes the results obtained for each patient that underwent the skin/BAT tests. The two patients that did not respond to the BAT were excluded from the comparison. Furthermore, Patient 2 and Patient 9 had previous episodes of severe reactions to skin tests, and for these reasons, they did not undergo those evaluations. Notably, among patients that displayed SI values > 2.5, Patient 10 underwent the COVID-19 vaccination with mRNA vaccines and developed injection site erythema.

## 3. Discussion

The administration of COVID-19 vaccines under medical supervision is needed in the case of suspected hypersensitivity reactions to the vaccines or their allergenic compounds (PEGs or PS80). It must be underlined in any case, that this is not a risk-free procedure, even if anaphylactic fatal events due to the COVID-19 vaccine administration could be considered very rare events [8]. For these reasons, regulatory agencies established specific guidelines for the safe administration of COVID-19 vaccines [1,23]. In such a context an accurate and timely diagnosis, based on reliable allergological tests, ideally sensitive enough to identify the effective patients at risk of developing allergic reactions, may be crucial. The efforts were mainly focused on PEGs, given that in Italy, as in many other countries, the previously approved viral vector-based vaccines were withdrawn from the market [24]. Over the last decades, the use of the BAT in allergological work-up programs has increased, given that it is a fast flow cytometry analysis, sensitive and highly advantageous for safety reasons [25]. It has been reported that SPTs are not useful in that context. Therefore, the last EAACI position paper, supported by other recent findings, underlined the utility of the BAT for the management of suspected hypersensitivity reactions to the mRNA vaccines [33,34]. For these reasons, we have implemented the use of the BAT in the allergological evaluations of 13 patients with a previous history consistent with PEG hypersensitivity or a reported allergic reaction to a prior vaccine containing PEG, or a polydrug hypersensitivity. Notably, we observed that, even if the BAT was negative for all tested patients, the SI calculation allowed the identification of a possible development of mild allergic reactions to PEGs (4 out of 11 analyzed subjects). The possible differences between the results obtained by the skin tests and the BAT may be related to the fact that the approved protocols do not require the use, as allergens, of PEG2000 and DMG-PEG2000 contained in the mRNA-based vaccines. 

As a matter of fact, whole mRNA and viral vector-based vaccines (i.e., Pfizer/BioNTech BNT162b2, Moderna mRNA-1273, Janssen/J&J Ad26.COV2.S and Oxford/AstraZeneca ChAdOx1-S) have been also used to test basophil activation in vitro, and it was demonstrated that preference should be given to mRNA vaccines or modified compounds containing PEGs over unmodified PEGs, which less often leads to positive results [25]. For these reasons, the stimulation with appropriate concentrations of mRNA vaccines could be useful to better clarify immediate reactions to COVID-19 vaccines.

Apart from the restricted cohort of enrolled patients, which is the main limitation of this study, which reflects the low frequency of adverse effects registered during the COVID-19 worldwide vaccination campaign, our data show that the BAT to PEG is a sensitive tool to be coupled with skin tests to identify PEG allergies, and by the evaluation of the related BAT stimulation index, we demonstrate, for the first time, that it is possible to identify eventual low-grade reactions even when the BAT results are negative.

In conclusion, we confirmed that severe hypersensitivity reactions to mRNA vaccines to COVID-19 are very rare, and their over-diagnosis must be avoided to ensure a complete and efficient vaccination for future campaigns based on vaccines containing PEGs. Therefore, the allergological work-up program for the patients reporting reactions after the vaccine administration is crucial to achieve a precise diagnosis. In that context, we demonstrated that BAT is a promising tool for the diagnosis of hypersensitivity reactions to PEGs and therefore its implementation in the allergological workup may be useful for the correct management of patients at risk to develop mild or severe reactions. It will also help to avoid public distress and the loss of confidence in vaccination safety, in the case of possible future new booster COVID-19 vaccine doses (i.e., against novel virus variants).

These findings will open new routes for the beginning of an era of personalized vaccinology in which we can tailor the safest and most effective vaccine on an individual and a population level.

## 4. Materials and Methods

### 4.1. Patients

About 800 patients with a history of severe drug reactions, including vaccines, underwent allergy counseling, as established by the shared protocol for allergic patients in the Chieti-Lanciano-Vasto ASL. The allergological work-up program started with a comprehensive clinical history, and skin prick test (SPT) and drug intradermal test (IDT) administration following the procedure explained in the SIAAIC-AAIITO consensus document https://www.asst-garda.it/wp-content/uploads/2021/05/Allegato-1-Documento-COVID_AAIITO_SIAAIC_15.02.21_compressed.pdf (accessed on 9 February 2021). Among all examined subjects, 61 patients with suspected PEG hypersensitivity underwent cutaneous SPT and IDT. Reactions were classified as grade 1 (mild: skin and subcutaneous tissues); grade 2 (moderate: symptoms suggesting respiratory, cardiovascular, or gastrointestinal involvement); and grade 3 (severe: hypoxia, hypotension, or neurologic compromise) [35]. Among patients that underwent the skin tests, compliant subjects with the most suggestive clinical features for PEG allergy reactions (N = 13) were enrolled together with 10 healthy volunteers from the Allergy Unit of the “SS Annunziata” Hospital of Chieti in 2020/2021. In detail, patients 1, 5, 10 and 12 were referred for previous allergic reactions to mRNA anti-COVID19 vaccines. Patients 2, 3, 4, 6, 7, 8, 9, 11 and 13 had a suggestive history of previous allergic reactions to drugs. More specifically, patients 9 and 13 were referred for a previous allergic reaction to other vaccines, while patients 4 and 7 had a previous reaction to drugs containing PEGs; patient 6 had a history of allergy to anesthetics and patients 2, 3 and 11 were referred for previous polydrug allergies. For each donor, a peripheral blood sample was harvested following the Ethical Committee approval number N.19 of 09 September 2021. Written informed consent was obtained for all recruited subjects (following point 32 of the Declaration of Helsinki 2013). The demographic characteristics of the enrolled subjects were reported in Table S4. No patients included in our study had dermatographism or any other disease that could interfere with the assessment of the response to the skin tests. None of the recruited healthy donors revealed any history of allergic clinical symptoms either to vaccine compounds, such as PEG or polysorbate 80, or to the vaccine itself.

### 4.2. Skin Prick Tests and Intradermal Tests

In the diagnostic workup, the skin prick test was carried out using a commercial undiluted drug (Depomedrol 40 mg/mL), containing methylprednisolone acetate, as the active compound and PEG 3350, as an excipient. When the SPT produced a negative result, three subsequent IDT were administered, at the following concentrations: 1:1000 (0.04 mg/mL), 1:100 (0.4 mg/mL) and 1:10 (4 mg/mL); only when the previous result was negative, the following was administered. The control test was carried out using methylprednisolone sodium succinate (Urbason 40 mg/mL), which does not contain PEG 3350. A wheal surrounded by erythema, with a negative response to the control saline solution was the criterion to judge the SPTs as positive, while an increase of at least 3 mm diameter of the initial wheal area surrounded by the erythema and the parallel negative response to the control drug was assessed as the criterion for the IDT’s positivity.

### 4.3. Basophil Activation Test

The BAT was carried out using the Flow CAST^®^ kit (Buhlmann Laboratories, Schonenbuch, Switzerland), using PEG2000, PEG4000 and DMG-PEG2000 as allergens. These molecules were provided by Buhlmann as highly purified allergens, CE-IVD grade products useful for diagnostics. For each tube, 50 µL of whole blood (collected using EDTA as an anticoagulant), diluted in 100 µL of stimulation buffer, was incubated with 50 µL of PEG2000 (final concentration of stimulation: 910 µg/mL), PEG4000 (final concentration of stimulation: 910 µg/mL), or DMG-PEG 2000 (final concentration of stimulation: 1.8 µg/mL; Flow CAST^®^ kit, Buhlmann Laboratories, Schonenbuch, Switzerland). The samples were stained using an anti-CCR3-Phycoerythrin (PE)-conjugated antibody and an anti-CD63- fluorescein isothiocyanate (FITC)-conjugated antibody (Flow CAST^®^ kit, Buhlmann Laboratories, Schonenbuch, Switzerland) and incubated for 15 min at 37 °C in a water bath after which the samples underwent a red blood cell lysis step (10 min, RT); finally, the samples were centrifuged for 10 min at 400× *g* and resuspended by adding 300 µL of wash buffer before the acquisition. As a negative control, 150 µL of stimulation buffer was used, and 50 µL of anti-FceRI monoclonal antibody and 50 µL of fMLP were used as positive controls. The samples were finally analyzed on a FACSCanto II flow cytometer (Becton-Dickinson Biosciences, San Jose, CA, USA), acquiring at least 500 basophils per sample (in the CCR3+ gate). The results were analyzed using FlowJo™ v10.8.1 Software (Becton-Dickinson Biosciences, San Jose, CA, USA), and activation was analyzed by both evaluating the frequencies of CD63+ basophils and calculating the stimulation index (SI) based on CD63 expression, as already reported [28]. Briefly, the SI was obtained by dividing the percentage of activated basophils (CD63+/CCR3+ cells) gated in the PEG stimulated sample by the basophil percentage gated in the related negative control. The BAT was considered positive when the percentage of activated basophils was higher than 6%, 5% and 6.5%, for PEG 4000, PEG2000 and DMG-PEG2000, respectively. Instrument performances, data reproducibility and fluorescence calibrations were checked by the Cytometer Setup & Tracking Module (Becton-Dickinson Biosciences, San Jose, CA, USA) [36]. CompBeads (Becton-Dickinson Biosciences, San Jose, CA, USA ) and the antibodies used in the assay were acquired to assess fluorescence compensation, while fluorescence minus one controls (FMO) were used to evaluate non-specific fluorescence [37].

### 4.4. Statistics 

Statistical analyses and descriptive statistics were carried out using GraphPad Prism ver 9.0 (GraphPad Software Inc., La Jolla, CA, USA) and XLSTAT 2022 (Addinsoft, New York, NY, USA). Statistical differences were calculated using the Student’s *t*-test as appropriate. A *p*-value < 0.05 was considered statistically significant.

## Figures and Tables

**Table 1:** General characteristics of patients and hypersensitivity reactions to PEGs.

Patients	Sex	Age	Skin Test	BAT
Patient 1	F	35	−	−
Patient 2	F	55	*	−
Patient 3	M	69	−	−
Patient 4	M	32	+	− (SI > 2.5)
Patient 5	F	52	−	−
Patient 6	F	50	−	− (SI > 2.5)
Patient 7	F	54	−	−
Patient 8	M	50	−	−
Patient 9	F	53	*	− (SI > 2.5)
Patient 10	M	36	−	− (SI > 2.5)
Patient 11	M	60	+	−
Patient 12	F	51	−	NR
Patient 13	F	54	−	NR

NR = non-responders; (NR): patients that did not display any significant activation when stimulated with the positive control samples. * Patients who did not receive the skin tests, because of a medical history of previous severe reactions to the skin tests.

## Data Availability

The authors declare that the data supporting the findings of this study are available within the paper and its supplementary information files. All datasets generated and analyzed are available from the corresponding author upon reasonable request.

## Supplementary Materials

The following supporting information can be downloaded at: https://www.mdpi.com/article/10.3390/ijms232314592/s1.
