# Supplementary material for: Basophil Activation Test with Different Polyethylene Glycols in Patients with Suspected PEG Hypersensitivity Reactions
Source: Int J Mol Sci. 2022 Nov 23;23(23):14592. doi: 10.3390/ijms232314592 (PMC9740962; doi:10.3390/ijms232314592)
Supplement: Supplementary file 1 [file ijms-23-14592-s001.zip › ijms-1976490-supplementary.pdf]

Table S1. Percentages of activated basophils (CCR3+/CD63+ cells). Patients (N=13) and healthy volunteers (Controls, N=10) were listed and the percentages of activated basophils upon PEG activation were reported together with the data related to the negative and positive controls (FcεRI and FMLP).						
	% CCR3+/CD63+					
**Cohort**	**PEG4000**	**PEG2000**	**DMG PEG2000**	**Negative Control**	**FcεRI (Positive Control)**	**fMLP (Positive Control)**
Patient1	0.65	0.45	1.62	0.78	87.4	39.6
Patient2	0	0.11	0	0.11	86.3	14.9
Patient3	0.23	0.22	0	0.12	85.5	25.6
Patient4	0.94	1.11	0.59	0.44	88.6	63
Patient5	0.57	0.68	0.46	0.8	50.8	1.44
Patient6	0.23	0.34	0.35	0.12	86.4	16.5
Patient7	1.59	1.32	0.9	1.14	77.6	12.2
Patient8	0.23	0	0.11	0.11	85.7	10.7
Patient9	0.5	0.69	1.16	0.23	93.7	26
Patient10	0.48	0.48	3.61	0.36	90.4	40.4
Patient11	0	0.11	0.11	0.23	66.9	59
Patient12	0.12	0.23	0.27	0.57	0.58	2.17
Patient13	0.5	0.85	0.13	0.58	0.74	3.66
Control1	0.46	0.47	0.57	0.88	87.7	60.8

Control2	0.34	0.91	0.55	0.68	61.2	21.4
Control3	0.47	0	0.12	0.26	93.4	0.24
Control4	0.5	0.55	0.16	0.44	92.4	18.6
Control5	0.34	0.11	0.34	0.45	18.1	13.5
Control6	0.12	0.39	0.5	0.36	80.7	57.4
Control7	0	0	0	0.53	69.7	17.4
Control8	0.00	0.00	0.23	0.34	92.6	43.3
Control9	0.12	0.12	0.24	0.11	87.7	15.7
Control10	0.30	0.14	0.14	0.37	90.2	24.8

Table S2. Values of activated basophils (% CCR3+/CD63+ cells), Stimulation Index (SI), % Basophils, and % Lymphocytes for analyzed patients (N=11) and healthy volunteers (Controls, N=10). BAT test non-responders were excluded from the analysis.

	% CCR3+/CD63+						Stimulation Index					
**Descriptive Statistic**	**PEG4000 Controls**	**PEG4000 Patients**	**PEG2000 Controls**	**PEG2000 Patients**	**DMG PEG2000 Controls**	**DMG PEG2000 Patients**	**SI PEG4000 Controls**	**SI PEG4000 Patients**	**SI PEG2000 Controls**	**SI PEG2000 Patients**	**SI DMG PEG2000 Controls**	**SI DMG PEG2000 Patients**
**Nbr. of observations**	10	11	10	11	10	11	10	11	10	11	10	11
**Minimum**	0.000	0.000	0.000	0.000	0.000	0.000	0.000	0.000	0.000	0.000	0.000	0.000
**Maximum**	0.500	1.590	0.910	1.320	0.570	3.610	1.808	2.174	1.338	3.000	2.182	10.028
**Median**	0.320	0.480	0.130	0.450	0.235	0.460	0.639	1.395	0.456	1.158	0.662	1.000
**Mean**	0.265	0.493	0.269	0.501	0.285	0.810	0.696	1.319	0.592	1.417	0.766	2.204
**Standard deviation (n-1)**	0.192	0.461	0.303	0.421	0.197	1.063	0.555	0.825	0.548	1.004	0.615	2.989

**Descriptive Statistic**	**% Basophils Controls**	**% Basophils Patients**	**% Lymphocytes Controls**	**% Lymphocytes Patients**
**Nbr. of observations**	10	11	10	11
**Minimum**	0.440	0.260	34.900	15.000
**Maximum**	1.450	1.380	61.400	60.700
**Median**	0.865	0.480	51.450	40.700
**Mean**	0.880	0.705	50.450	40.382
**Standard deviation (n-1)**	0.381	0.437	9.394	12.727

Table S3. Statistical differences between controls and patients analyzed.	
**Variables**	**p-values (Controls *VS* Patients)**
% CCR3+/CD63+ PEG4000	0.164
% CCR3+/CD63+ PEG2000	0.168
% CCR3+/CD63+ DMG PEG2000	0.141
SI PEG4000	0.059
SI PEG2000	**0.033**
SI DMG PEG2000	0.153
% Basophils	0.341
% Lymphocytes	0.055

Statistical differences were calculated by Student's t-test. A p-value of <0.05 was considered statistically significant. BAT test non-responders were excluded from the analysis

Table S4. Demographic characteristics of the study participants.				
**Cohort**	**n**	**Age (years) [Mean±SD]**	**p-values**	**Age (years) [Median]**
**CTRLs**	10	37.60 ± 9.06	0.006	40
**Patients**	13	50.08 ± 10.32		52
**Gender**	**%**	**Age (years) [Mean±SD]**	**p-values**	**Age (years) [Median]**
**Male**	34.8	44.00 ± 15.05	P>0.05	41.00
**Female**	65.2	45.00 ± 9.651		50.00
